# Dental Patents

**Published:** 1853-07

**Authors:** Robert Arthur


### ARTICLE II.

*Dental Patents.* By ROBERT ARTHUR, M. D., D. D. S.

AN article bearing the title of "Dental Patents" appeared in the last number of the "Journal of Dental Science." Well written and plausibly argued, it is, certainly, the strongest defence of the practice of protecting discoveries or inventions in our profession by patent-rights, which has yet appeared; although, indeed, but little has been said on this side of the question. The tendency of this article I cannot but regard as highly injurious; and I shall endeavor to show that the arguments of which it is made up are not, by any means, firmly based. In discussing this question as Dr. Hill has presented it, and in its general aspect, I shall be very careful to avoid remarks which can be regarded as personal, except so far as, in the case of Dr. H., he necessarily identifies himself with the opinions he advocates, and in that of others, they are forced by circumstances, into personal notice. I cannot allow this opportunity to pass without saying, that I disapprove most decidedly, of all public personal controversies, and will not be induced to engage in a discussion which can be allowed to take anything of a personal character, especially in a publication devoted, as this is, purely to professional objects.

I agree with Dr. H. when he says, that upon this subject, as, indeed, I may add, upon almost every one connected with our profession, a great many high-sounding, unmeaning words have been expended; and I shall therefore endeavor to examine the positions taken by Dr. H., and the general question, as briefly as may be consistent with an adequate presentation of the subjects involved.

Dr. H. contends that a dentist who may happen to make any new discovery, or invent any thing which may be used, with advantage, by his profession, has, not only a legal and a moral, but, also, a professional right to obtain the control over it, and the profit arising from it, which a patent will give him; and he complains, bitterly, of the course of the profession toward those who have thought proper to pursue this practice, characterizing the opposition which has been made to it, as illiberal, unjust, and springing from selfish motives.

It is necessary that I should state, at the outset, that I have nothing to say against the general application of the patent-laws out of our own profession; and the opposition I am disposed to make to their application to our profession has reference to such objects only as have a strict bearing upon its immediate interests. I do not, of course, regard their application to the medical or other professions, as any more admissible, but they are quite able to take care of their own interests in all respects.

The subject of patents, then, as it has a strict relation to our own profession I propose to discuss.

The legal right is, of course, not disputed; the moral right is by no means so clear. A great deal might be said upon this point, but it is one which I have not now time to examine. But if in the course of this argument it is not incidentally shown, that this right does not exist, I hold myself ready at any time to go at greater length into its discussion.

I propose, then, in this paper, to discuss the professional right, upon which Dr. H. has boldly thrown himself. By professional right I understand Dr. H. to mean the right to pursue the course indicated, without thereby incurring the censure, or even the slightest disapprobation, of the liberal-minded amongst his fellow practitioners.

I will now follow as nearly as I can Dr. H's line of argument.

He first satisfies himself, with great facility, that he has a moral as well as a legal right to cover his discovery or invention with a patent, and then, for the first point in his argument, declares it impossible that the legal and moral right can exist without the clearest professional right. If he had clearly established the moral right of the course for which he is contending, this position would have had more strength; but this he certainly has not done; and the question in its best aspect for him, remains open. I will proceed, then, to examine the grounds upon which he claims the professional right.

In the first place, Dr. H. takes the position, that the taking out of a professional patent, if a fault at all, is only a venial one, as it is a sin against a mere conventionality, and does not merit, by any means, the severe condemnation with which it has been visited on all sides. But even this slight offence he declares has not been committed by the class of individuals alluded to; for, defining a conventionality as an agreement formally entered into, or in spirit, a tacit understanding arising out of long usage, he denies that, in our profession, there has been any such formal agreement, or any such tacit understanding, resulting from long usage.

But Dr. H. has certainly mistaken one of the true grounds of objection to this practice. It is not on the ground of mere conventionality (although there *is* a tacit understanding adverse to it amongst the better class of dentists, which certainly deserves this name) that this practice is most strongly objected to; nor can the general repugnance now felt and exhibited toward it be called a "hoary error." This strong feeling is of comparatively recent date, and has gradually grown up from experience of the bad consequences resulting from it and similar practices, for many years.

It cannot be questioned that our profession has made great advances in the last few years. It has advanced in knowledge of general principles, and in their application to practice. It is also a fact, recognized by every intelligent man, acquainted with the history of our profession, that the rapid progress it has recently made is, mainly, attributable to the perfectly free and unrestrained interchange of ideas and opinions which has, for the last few years, prevailed.

It is well known, too, that, until recently, (certainly within the recollection of some who are not by any means the oldest now amongst us,) our profession has dragged out a slow and miserable existence; and it cannot he denied that, in a very great degree, its condition in this respect, is attributable to the very narrow spirit which induced each individual to keep closely to himself everything which he may have thought or done.

All the older, and many of the comparatively younger members of the profession, well remember the many obstacles which they encountered, and the many sickening difficulties with which, from the cause above alluded to, they had to contend, in the earlier part of their career. They found it absolutely impossible, except in rare cases, to obtain any kind of assistance from the experience of those who had gone before them in the same path over which they were traveling. They longed for association with men engaged in the same pursuit, that they might learn something from their experience which would assist them to solve the difficulties that daily presented themselves in practice. They felt that, in many cases, they were groping in the dark; they were dissatisfied with what they accomplished; they believed that more could be done than they were capable of doing; and whilst they were willing, themselves, to labor to obtain better results, and were ready to share freely with others the advantages of such results, they felt the necessity of, and desired, all the assistance they could obtain. But to those who were in advance of them in the profession, there was no access; every door was closed against them; and if, by accident, they came into contact with these gentlemen, the profession in which they were mutually interested, was a sealed subject. Even amongst those whose age and experience placed them upon the same level, there was no professional intercourse, nor, indeed, with rare exceptions, intercourse of any kind.

Some of these persons had paid dearly for the modicum of knowledge of dentistry which they possessed. Experience had shown them that they must pay dearly for what they learned from others; and they evidently regarded their knowledge, either purchased from others, or picked up by themselves, as their own property, worth to them, like other goods and chattels, so much money.

Many of them, too, were men who had made a great advance from their former position and condition in engaging in the pursuit of dentistry, low as it then was; and, finding that they had fallen upon much more lucrative employment than that to which they had been accustomed, with the natural but erroneous instinct of selfishness, they wished to prevent others entering upon the same field, from the apprehension that they would be deprived of something which they might otherwise appropriate to themselves. For this reason they could not, for any consideration, be induced to instruct others in what knowledge they had of the profession, except those in whom they were strongly personally interested, or those who would bind themselves, under heavy obligations, not to practice in the same vicinity, or where there could be any possibility of conflict. It is stated, (on good authority, too,) that the stipulation, was once made, by a man occupying, at the time, a high place in the profession, that a student with him, after he had been instructed, was not to practice in the same city, and was not to teach any students; the preceptor, at the same time, binding himself that he would not instruct any one else to go to the same city, which was a very large one, needing the services of many more competent dentists than it even now contains, although some years have elapsed since this transaction took place.

In many cases, too, where, for a consideration, some of these persons took students, it was to give them only a partial knowledge of what they knew themselves. For one or two hundred dollars, more or less, they would, perhaps, allow them to remain in their workshops for a few months, place in their hands such works on the subject as were then extant, (of the practical value of which it is not necessary that I should say anything,) *tell* them how various operations were performed, let them see some of the instruments they used, and then, after the fee was paid, give them a certificate of competency to practice where there was no possibility of conflict with themselves; at the same time guarding closely, or selling for an additional sum, all that was peculiar to themselves, or all which they regarded as of particular importance.

Service, more or less valuable, was rendered by dentists, at the time to which I am alluding; although, with the exception of what was done by a very few practitioners, such service was confined to the more simple operations, or, when more was attempted, it was not attended with any beneficial results. It was soon discovered that dentistry was an occupation which would yield a larger return for the investment of a smaller amount of capital and knowledge than most others. The inevitable consequence was, that the demand was supplied in some fashion. Many individuals who failed to succeed in other employments, turned to this without the slightest preparation, and the profession became crowded with the most ignorant and inefficient candidates for employment. This necessarily proved to be an additional reason for an entire absence of anything like professional intercourse.

But amongst the large number employed in the practice of dentistry, there were some who turned toward it with the right feeling, actuated by proper motives. Whilst they looked to the profession for support, they desired to do what good might be in their power, and spared no pains or labor to prepare themselves, thoroughly, so far as they knew what was required. Many of these men felt a consciousness of their ignorance and inefficiency, and longed for more knowledge; but there were no means of increasing their stock, except by their own unaided efforts. I was particularly struck and affected, at the time, with the account of such a man as I am now describing, of his own aspirations for knowledge and the chilling obstacles of the kind which he encountered. The allusion made will be readily understood by those who were present on a recent festive occasion, where there was a great deal said about the past and present condition of our profession and its prospects. The gentleman alluded to, who now occupies a prominent place in the front rank of our profession, was recalling the troubles of his early practice. "I longed," said he, in substance, "for some kind of open, frank intercourse with intelligent men of our profession. But I was acquainted with the strong feeling of exclusiveness which prevailed amongst most of those who could, in any way, be worth seeking. I was young and unknown, and feared to meet with a repulse if I called upon any of them. But I felt my own ignorance; I knew that I was an earnest seeker after knowledge, and I was anxious to contribute what I could toward the advancement of my profession and all who were engaged in it. I saw there was some reason for the feeling of exclusiveness which prevailed, but I longed to see it extinguished, and believed that there must be some who would meet, in the right spirit, my advances. With this purpose in my heart, I have many times walked back and forth before the door of a dentist of standing, longing to go in and offer and ask for a generous freedom of intercourse, and when, at last, with this earnest object in view, I did so, I met with nothing but a cold repulse."

But these young men grew older; with strong wills, clear heads, apt hands, they not only advanced, themselves, in time, to the point occupied by those with whom they sought association, they passed them, and developed new capabilities and new resources of our profession not before conceived of. I am now stating what cannot be successfully denied, however gentlemen may wrap themselves up with a sneer, in their mantle of self-sufficiency; and I hazard nothing in saying that the majority of those who, at the present time, occupy, deservedly, the most prominent places in our profession are men of precisely the character I have been describing.

They soon discovered each other—they came together on all fitting occasions—they compared views—they interchanged ideas—they could not fail to discover that this was the most certain means of progress, and they set their faces, decidedly, against everything which could at all tend to interrupt it. They could not fail to perceive that the selfish feeling which induced men to endeavor to appropriate to themselves all the pecuniary benefit they could possibly derive from their discoveries, would be an effectual bar to all liberal intercourse. It is in this way that the spirit complained of originated. It is this spirit that I regard as laudable, to be warmly cherished, for to it is due most of the vitality our profession now has, and with its extinction would die out its most beautiful and attractive qualities.

Is it surprising that men who have gone through this experience, of which I have felt compelled to say so much, who are deeply interested in their profession, and see, so plainly, the true grounds upon which its future prosperity depends, should feel strongly and express themselves strongly, in relation to this matter? Is it strange that they should feel a deep disgust of everything which, in the remotest degree, tends to bring the pursuit in which their best and warmest feelings are engaged, back to the degraded depths from which it is still struggling to emerge, just at the moment when it gives good promise of taking the position it so well deserves to occupy?

It may be said that all this is irrelevant; that it is wandering, a great way, from the subject in hand; that there is no question about the impropriety of making secrets of what each man may do or learn in his profession. It may be denied, and it has been, that the protection of a discovery by a patent does, in any way, interfere with the freest interchange of ideas amongst members of any profession. It may be said that the discoverer or inventor is obliged to disclose his idea fully, let it be of whatever character it may, before he can obtain the benefit of the patent laws; and that this is a very different thing from keeping secret his discovery for his own exclusive benefit, or for the purpose of selling it to those who may wish to make use of it. Now the two practices can be defended on precisely the same ground. If one is necessarily right and proper, so is the other. If one is wrong and injurious, so is the other —they are unquestionably involved in the same category. The right of a man in our profession to patent an invention or discovery is contended for on the ground that it is his own property, and that he has, therefore, the right to secure to himself the benefits resulting from it in the best possible way. Covering a claim to exclusive right in an invention or discovery may not always be so effectually done by means of a patent as by keeping a process secret and selling the particular article which has been produced; because, when the discovery becomes fully known, it may, in the large majority of cases, be used without regard to a patent, and no pecuniary return may be reaped from it. It is well known that Swaim acted upon a knowledge of this fact, and instead of procuring a patent for his panacea, which yielded so large a fortune, it was and is, manufactured secretly. Indeed, the very worthlessness of dental patents, at the present time, of which the proprietors complain so bitterly, will legitimately lead to this result where it is likely to be more available to keep secret a process than to cover it with a patent. And if the right of the inventor who acts in this way to perfect respect and good will of his brother practitioners be fully admitted, it is not difficult to see the end to which it will lead.

Let us trace the legitimate consequences of a general admission of the right and propriety of this practice of taking out patents for every improvement in the profession: and I do not see how these can be separated, for, if a thing is strictly right in itself I am unable to see how it can be improper. It must be evident that if the practice is sustained, it is applicable to every invention, discovery, and improvement of every kind. No standard, other than the law, can possibly be set up, by which the right and expediency, of patenting an article may be ascertained. It will not do to say that it is inventions of more importance only, or those which are made at great cost of time, money and labor, that are worthy of this protection. What tribunal is to decide upon the value of anything new? Any individual to whom a new idea occurs, however trifling it may be in the estimation of other men, may regard it as of paramount importance. In our own profession it cannot, I think, be denied, that the state of mind which would lead to such a conclusion is a very common weakness. I do not know any class of men, so far as my intercourse with them has gone, who are so well satisfied with themselves, and who estimate more highly everything which they originate. If the right, then, were generally admitted, and generally countenanced, and generally put into practice, as it might be, there would be a patent for every new curve or point given to an instrument, for every tooth-powder and mouth-wash which any individual might concoct. Or if a patent were found or supposed to be insufficient to protect the invention where it happened to be of such a character as would make this course available, it would be kept secret, and the right of making use of it sold at any price the discoverer might be disposed to set upon it, just or oppressive, if necessary to the profession, in proportion to his sense of right or his cupidity.

Imagine a meeting of a dental association regulated upon such a basis as this. If there could be sufficient good feeling to make it possible to obtain a meeting of dentists, under such circumstances, at all, what would be the amount of value of their intercourse? It would be simply a means of advertising their improvements—bringing them to the best market. It is unnecessary to dwell upon this point; it is not at all difficult to see the end; it is, inevitably, the breaking up of all general association and its ruinous consequences.

The same effect, too, must inevitably occur with regard to personal intercourse. What satisfaction will it afford any man engaged in a particular calling, to meet with another pursuing the same way in life, if his lips are obliged to be sealed upon the subjects which must necessarily occupy the largest place in his thoughts and interests? This picture, it may be said, is overdrawn; the inferences, it may be said, are violently strained. But I do not see how this can appear to any man who will carefully weigh what I have said, unless his mind has already taken a decided bias in another direction. I have shown that a similar state of things has already, from the same cause, existed in our own profession; and I am unable to see any just ground for regarding this as an exception to the rule, that like effects follow like causes.

Look at the condition of society around us. Look at men engaged in the same pursuits, except in those cases where their views have been so expanded and liberalized by proper cultivation as to enable them to see, more clearly, their true interests. See how carefully the shops of most kinds of manufacturers are kept closed against all visitors, and particularly against those who are engaged in the same pursuits. Observe how very carefully particular processes are kept secret. You will see the proprietor of an establishment quietly retiring to his private room, locking himself in, and making the little compound, or dropping in the particular chemical, which is the important link in a chain of results. In business and manufactures, generally, this, I believe, is regarded as perfectly justifiable. It may be so. But if it is, I doubt very much the policy of such a practice. It is my firm belief, that in every pursuit, if men would freely compare their ideas, and their experience in working them out, every individual would be more benefited by it, and much larger advances made towards perfecting processes of every kind.

But it is contended that in setting our faces so decidedly against such practices, great injustice may be done; and the strong picture has been drawn of a man who has been led "into a series of experiments involving time, labor and expense, and eventuates in the discovery of a process by which his professional brethren can be greatly advantaged, and immense good to mankind result from it; meantime, in perfecting and bringing his discovery before the world, his means are all exhausted—his family destitute, and he greatly embarrassed with debt, and that debt incurred in prosecuting his experiments. But, with the devotion peculiar to this class of persons, he continues, and by 'hook or by crook,' he completes the invention. The thing is done—he is bankrupt. What is his duty under the circumstances?"

Now, in such cases as this, to deprive a man of a fair remuneration for what he has done at so great a sacrifice would certainly appear unjust and cruel. But there is one principle upon which such a course can be fully justified; it is this: all laws, however good and useful they may be, do more or less injustice, under certain circumstances, to individuals; but the general good must be consulted and preferred when it comes into conflict with private interests. I have shown, clearly, I think, that if the right of the practice of protection by patent of professional discoveries relating to dentistry were universally admitted and justified, it would result in great injury to the community and to the profession; such a case, therefore, as is above instanced by Dr. Hill might occur without at all weakening the position I have taken.

But a great deal more may be said upon this point. The method indicated, is not the only one by which a man may be remunerated for such services, and particularly in our own profession.

I have nothing to say about the "glory of a noble act," &c., being sufficient return for such a sacrifice.

It cannot be denied that there is a great deal of selfish disregard of the interests of others in our own profession, as well as in the world, and many persons will always be found ready to do injury to the best and most deserving men, if it will, in any way, lead to their own profit or advancement. But in all communities there is a sense of justice with some, and a sense of shame with others, which generally places credit and profit where it belongs. And if any case similar to that imagined by Dr. H. could actually occur, I have so much faith in the justice and liberality of the better members of the profession, as to believe that they would make some provision for it. And with regard to the only actual case which has any resemblance of the kind, however faint that may be, and which has been alluded to by Dr. Hill, (I mean that of Dr. J. Allen,) the course of the American Association of Dental Surgeons, proves that I have some foundation for this belief. Allusions have, from time to time, been made to this case as one of flagrant injustice, in some respects; and I think the facts of the conduct of the profession toward Dr. A. cannot be too frequently or distinctly stated. The better class of dentists, who have always most strenuously taken ground against this professional patent practice, were not only far from unwilling, but were anxious to make some immediate return in a proper way, for the time, money, and labor alleged by Dr. A. to have been expended by him in perfecting his improvement.

I remember, with great distinctness, all that transpired in relation to this matter, at the time it was introduced to the profession. I have no question that Dr. H. will be able to recal the same facts, as he was present on the occasion. It was at the meeting of the American Association of Dental Sergeons, held in August, 1851, in Philadelphia. Dr. A. exhibited his improvement, which was remarkably beautiful and striking. It is well known that the spirit and express laws of this association are opposed to everything which can, in any way, interfere with the freest interchange of ideas, generally, and, if I mistake not, to professional patents, particularly. Dr. A. was immediately asked, by a gentleman, who, in open meeting, rose upon the floor for the purpose, what were the ingredients of the composition, and what was the process by which he accomplished the results obtained. Dr. A. replied, in substance, that it was impossible to make a statement which would enable any one, practically unacquainted with the process, to avail himself of its advantages, even if he were acquainted with the ingredients employed. That he had spent a great deal of time, and money, and hard labor upon the improvement, and he thought it only just that he should reap something in the way of remuneration for his efforts. He had lodged a caveat in the patent office to save himself from being overreached, but it might lie there forever. He was opposed to patents in our profession, but thought it only just to demand some return for his time, labor, and money. This he proposed to obtain, (and the course, he presumed, could ot certainly be objected to,) by giving personal instruction, for a reasonable sum, in the manner of applying his improvement. (See Journal, new series, vol. ii, p. 165.)

A vote of commendation was immediately passed, and several members of the association—although they were at the time aware that the right of discovery was contested by Dr. Hunter of Cincinnati, and that that gentleman had declared his determination to give the particulars of the whole process to the profession—applied to Dr. Allen for instruction, and were, I know, willing to pay him liberally in this way for the improvement he seemed to have made. Dr. A. was not prepared at the time to give the desired instructions. Now, what was the subsequent course of Dr. A.? In the face of this assertion before the association, "that he did not approve of professional patents," we find him declaring before the "Mississippi Valley Association," one month afterwards, that it is "essential to the advancement of the profession that we recognize the right to patent improvements." And he proceeded at once to procure a patent. The very men who applied to Dr. A. and were willing to pay liberally for the use of his improvement, would not subsequently touch it at any price. And is it to be charged that great injustice has been done to this person by discountenancing his course? Can it be claimed that such conduct as this deserves the highest respect of the profession? For one, I am not, and never shall be, willing to accord it.

But the case imagined by Dr. H. is, I contend, one which is not likely to occur. It may possibly happen; but a thing of this character which has never occurred in our profession, may certainly be supposed as not very likely to occur. In the long history of our profession there is no case recorded of a man who has impoverished himself by investigations, (pursued in the strict line of the duties of his vocation,) which have led to any discovery important or unimportant. Even if he may have devoted a large amount of time, labor, and money, to the improvement of his profession, whether he is successful or not in developing anything strikingly new, he will, I am satisfied, be benefited in every way by his efforts. I know that no man in our profession has been ruined in this way, and I very much doubt whether the pecuniary fortunes of any one have been damaged by such a course. On the contrary, I believe that the effect will be exactly the reverse; for if his investigations be judiciously directed, the result will unquestionably be an increased knowledge of his profession, increased reputation, and a necessarily increased demand for his services. If he succeed in making any discovery of consequence, the eclat which attends it will generally bring reputation and a pecuniary return much more than equivalent to the value of the time and labor which he has directed in the new path. The very discovery he has made, too, will itself be another means of advancing his interests.

I have made careful inquiry of intelligent members of the medical profession in relation to this matter, and the universal reply is, that no such case as the one alluded to has occurred within their knowledge.

But it cannot be denied that many of the improvements applicable to our profession and valuable to it, have not been the result of long and laborious investigation, but have been the result of ideas occurring and developing themselves in the course of a regular and thoughtful practice. And many of these ideas have developed with infinitely greater rapidity in consequence of the light thrown upon them by other intelligent minds directed in the same way. I am sure the instance of the operation of the extirpation of the pulp, which has now reached a degree of importance (let this be questioned as it may) that renders it invaluable both to patient and practitioner, is one to which I may safely allude. What has been done in this way has been legitimately within the range of the profession, and has been the work of a number of individuals laboring toward the same end. It is well known, too, that what is now regarded, and is, the important feature in this treatment of the exposed pulp, (I mean the use of arsenious acid for first destroying its vitality,) was the discovery of a gentleman who freely gave it to his fellow practitioners.

Our profession, it cannot be denied, although a laborious one, makes a fair return for the labor it requires; and I do not see how we can strongly contend, to its injury, for our right of property in ideas occurring in its regular practice. And I think particularly applicable, in this connection, to practitioners of our own profession the sentiments expressed by Lord Bacon, in the preface to his Law Tracts: "I hold every man," says he, "a debtor to his profession; from the which, as men of course do seek to receive countenance and profit, so ought they, of duty to endeavor, themselves, by way of amends, to be a help and ornament thereunto. This is performed, in some degree, by the honest and liberal practice of a profession, when men shall carry a respect not to descend into any course that is corrupt and unworthy thereof, and preserve themselves free from the abuses wherewith the same profession is noted to be infected; but much more is this performed if a man be able to visit and strengthen the roots and foundations of the science itself; thereby not only gracing it in reputation and dignity, but also amplifying it in perfection and substance. Having, therefore, from the beginning, come to the study of the laws of this realm with a desire no less, if I could attain unto it, that the same laws should be the better for my own industry, than that myself should be the better for the knowledge of them."

Dr. H. strongly charges that selfishness, "like a serpent," lies at the bottom of all these attacks against the practice for the right of which he is contending. In one sense, but not the one intended by Dr. H., this is true. We are contending for our selfish interests. I believe, firmly, that by steadily opposing the growth of the spirit I am condemning, I am doing my part, for reasons I have already stated, to subserve the best interests of a profession in which I am deeply interested and upon which I am dependent for the subsistence of myself and family. I wish to be on terms of the freest intercourse with my fellow practitioners, because, whilst I am willing to give them all I may perchance learn or discover in my own practice, I expect to receive a large return from them. In exchange for my own ideas, if they are worth anything, or even if they are worthless, I receive those of a hundred others. I acknowledge, that in this respect, I consider my own interests; but at the same time I believe that it is, in the same degree, the interest of every individual engaged in our profession.

But when it is said that we wish to filch from others their hard earnings, I deny, indignantly, the charge. There is not a single patent relating to our profession, on the records of the patent-office, for the use of which I would, myself, give the smallest fraction of the smallest coin that has ever been issued. There is not now, and there never can be, a single discovery in our profession, encumbered in this way, of which I would make use, even if it were gratuitously offered to me, except for the simple reason that my patients would be injured by my refusal to do so. I know of none such at present in existence. And the charge of selfishness in this sense, comes, I feel obliged to say, with a bad grace from those who are willing to receive the advantages of the free offerings of those who are laboring well and faithfully in the cause of our profession, and yet, after they have been well repaid, in many ways, for all they have done, or can do, claim a miserable sum of money from every one who expects to reap the slightest advantage from anything which they may have wrought out.

There is one more point in the article alluded to, of which, before closing, I wish to take brief notice. It is the comparison drawn, with so much confidence of their perfect identity, between patent-rights and copy-rights. Although they may, to some extent, be placed on the same ground of right of property, there is, I conceive, a very material difference between them. So far as relates to our own profession there is certainly a very important difference.

It will be observed that I do not contest even the moral right of patents except so far as the practice applies to our profession, and then on the ground only, which I think I have shown, that it is injurious to its general interests. Now, if I could be convinced, that this practice might be generally adopted without injury to our profession, I should not have one word further to say, and would be far from disapproving of the course of those who choose to avail themselves of its advantages. When it can be shown that the practice of protecting with a copyright any treatise, upon subjects connected with our profession, is injurious to its interests, I will give up the whole question.

When a man writes a book he desires to have it published. Unless it is printed and published, it will not be of much value to anybody but himself. Now, beside the necessary capital to purchase paper, pay for printing and binding, all necessary, it requires certain business facilities to place the book where it will be accessible in all parts of the country, to those who may wish to purchase it. The author avails himself of the capital and business-facilities of the publisher to bring his book before the public. But the publisher will not risk his capital and give his time and labor without some reasonable certainty of remuneration; with this object in view, he requires the monopoly of the sale of the book in question. If he give it to the world without the protection of a copy-right it may be taken up, carelessly and cheaply printed and published by any one; and in many cases the publisher must either lose in his undertaking, or his profits must be very much diminished.* It is clearly the interest of the publisher and the author, however, that the book should be offered to purchasers at the lowest remunerative rate; and there is always a point in the price for which it is sold, which will yield the most money. The price may be so high as to lessen its sale so much as to cause loss; and it may be so low as to lead to the same result. This matter is well considered by the publisher, the price of the book is fixed at the point indicated, and the author receives a certain per centage on its sale, or sells out his right of property in it to the publisher. It is said that the author's tax on the book obliges the publisher to sell at a higher rate than he would otherwise do; but this cannot fairly be assumed, for, as I have said, it is the interest of both publisher and author to sell the book at a rate which will yield the largest return in money, and it is perfectly immaterial to the purchasing public whether the copy-right is vested in the publisher or in the writer. It is the clear interest, and it is the acknowledged right, and the invariable practice of business men to make all they can out of any enterprise in which they engage; and even if the author were to give his book to the publisher, there is no just ground for inferring that the purchaser would be in the slightest degree benefited by this sacrifice on the part of the author. For this clear reason, it seems to me that it cannot with any degree of force be contended, that the practice of covering his productions with a copy-right can be charged upon an individual as inflicting any injury upon our profession, because any sacrifice of this kind on his part would not necessarily increase the sale of his book, and give a freer circulation to his ideas.

* It may be objected here that this thing, notwithstanding, is constantly done; that large numbers of books are republished in this and other countries without copy-right. But it is scarcely necessary to say that these are, generally, if not invariably, books which have a large and rapid sale, or are works of such magnitude or character, that when the market is once supplied no other publisher will incur the risk of publication.

There may, also, be this distinction between a copy-right and a patent-right relating to our profession. Many of the more valuable discoveries have undoubtedly been the result of accident and many of the most valuable ideas have flashed upon men engaged in the regular pursuit of their business, requiring no effort, no labor to work them out. The proceeds of a copy-right are wages for labor actually performed, and it is generally very poor pay, too, except in cases where an author has great and striking abilities, and where his work meets with a necessarily large sale, as in the case of Macaulay instanced by Dr. Hill. In our profession, as there must always be a comparatively small sale of any work published, the compensation for the actual physical and mental labor performed must be very small. I have not the slightest question, if I may again venture to make a personal allusion, that the same time devoted by Professor Harris, to the practice of his profession, which has been given to the production of the works he has published, would have yielded him a much larger pecuniary return. It is not sufficient objection to say that this work was performed at a time which could not have been devoted to the practice of his profession; for after the heavy labor of the day, which a full practice demands, the residue of the time which is not given to necessary rest and recreation is doubly valuable. And I think that, instead of attempting to lessen the value of such service in order to bolster up a bad practice, the profession, beside giving freely (even if it came out of their pockets, which I have shown it does not) the pittance demanded in the way of pay for labor actually performed, owe a debt of good feeling which is not easily cancelled.

There is another difference between the patent-right and the copy-right which, when fairly examined cannot be considered a trifling one. For the sake of clearness, I will examine it in its relation to our profession, although it is very general in its bearing. The individual who purchases the right to make use of a patented article, or to follow a particular patented process in his own practice, cannot permit any one to obtain the same advantages which he derives from it, except by the express permission of the patentee, and the payment, if it is exacted, of a sum of money similar to that which he has paid for the privilege. The patentee may have made, with slight labor, some modification in an article of trifling value, which it may be of importance to a practitioner to use, and this he may sell at an exorbitant advance over its original cost. However cheap the raw material may be—however easily it may be obtained—however trifling the labor required for its preparation—the man who makes use of the article in question, must purchase from the patentee at the exorbitant price demanded, or subject himself to heavy penalties consequent upon a violation of the law. In this way the patentee reaps a pecuniary return exactly in proportion to the extent of the use of his patented production. Now let us turn to the other side; let us examine the copy-right by the same light here brought to bear upon the patent-right. What is the effect of the copy-right? I have clearly shown, I think, that it simply enables the author in our profession, to have his book published, and placed where it will be easily accessible to purchasers, and to obtain a small compensation for labor actually performed. I think I have, also, succeeded in showing that this compensation to the author does not come out of the pocket of the purchaser. Now, when the book is published it is accessible to every one, and, in the nature of things, at a low rate. It is impossible, at the present day, to put up the price of a book to an exorbitant height, for however highly the author might estimate his production—and however great his cupidity might be, he could not find a publisher who would afford him the necessary aid, unless he would, to a certain extent, allow him to regulate this matter. Here, then, is a great difference; the patented article is entirely independent of any third person. The patentee may exercise his own discretion in fixing the price he expects for his production; the author has not the same means of bringing his work before the public, and must do it through the medium of a publisher who regulates the price at which it must be sold. The inevitable consequence is that any book relating to our profession must be sold at a comparatively moderate sum. Now, whatever the book may contain is at the free disposal of the purchasers. If it sets forth new and important ideas, (and the most valuable discoveries have been given to the world in this way,) they are at the disposal of every man. The purchaser of the book is not only enabled to use these new ideas for his own advantage but he may teach them to as many others as he pleases. He may lend the book to any number of persons; or he may make himself perfectly acquainted with what is most valuable in it and sell it again at a trifling discount. He may take an abstract of its contents, glean from it or condense what is new and important, and publish this in any form he may please. He may even take the ideas which the book contains, put them in his own language, make a book and sell it on his own account; a thing not so very rarely done in these days. In this way, it must be plainly seen, that whilst the patent can be used by the purchaser only, a single book, even under the restriction of a copyright, may yield the same advantages to hundreds. Now, if the perfect identity of these two practices can be contended for in the face of this statement, it will be folly to expect men to perceive the most obvious truths.

Upon this branch and other branches of Dr. Hill's argument, and upon the whole subject, a great deal more might be said; but I have already occupied more time than I intended to give to it, when I began; but with all my desire to be brief, I have not felt that I could say less.

It will be observed that I have argued this question upon the selfish ground, that it is the interest of every one engaged in our profession to oppose the practice which has formed the subject of this paper. There are higher and nobler grounds than this, of which, from time to time, a good deal has been said, and of which much more might be said. But as such arguments appear at the present day to be regarded as smacking of mawkish sentimentality, I have judged it discreet to conduct my argument in such a manner that it could not fail to appeal to the understanding of every one.

